# Low dose of flurochloridone affected reproductive system of male rats but not fertility and early embryonic development

**DOI:** 10.1186/s12958-019-0508-5

**Published:** 2019-08-06

**Authors:** Rui Li, Su Zhou, Hongyan Zhu, Zhichao Zhang, Jing Fang, Ping Liu, Yu Wang, Xiuli Chang, Yubin Zhang, Liming Tang, Zhijun Zhou

**Affiliations:** 1 School of Public Health/MOE Key Laboratory for Public Health Safety/NHC Key Laboratory of Health Technology Assessment, Fudan University, Shanghai, 200032 China; 2 Pharmacology and Toxicology Department, Shanghai Institute for Food and Drug Control, Shanghai, 201203 China

**Keywords:** Flurochloridone, Reproductive toxicity, Fertility, Early embryonic development, Rats

## Abstract

**Background:**

Fluorochloridone (FLC) is a widely used herbicide, and its target organs are testes and epididymides. The Globally Harmonized System of Classification and Labelling of Chemicals classified FLC as Level 2-possibly cause fertility or fetal damage (no relevant data support). The maximum residue levels of FLC in processed crops have been reviewed in the latest European Food Safety Authority (EFSA) report in 2018. However, the toxic effect of FLC on fertility and early embryonic development is limited, and the health risk assessment of FLC needs further consideration. This study investigated the potential effects of FLC on fertility and early embryonic development in rats.

**Methods:**

One hundred rats of each sex were divided into four groups including three FLC-treated groups at doses of 2 mg/kg, 5 mg/kg and 15 mg/kg, and a vehicle control group (0.5% (w/v) sodium carboxymethyl cellulose). Male and female rats were dosed for 9 and 2 consecutive weeks, intragastrically, prior to cohabitation and lasted throughout the mating period for males and continued until Gestation Day 7 (GD7) for females. Parameters such as weights and coefficients of reproductive organs, epididymal sperm number and motility, indexes of copulation, fecundity and fertility indexes, mating period, estrous cycle, corporalutea number, implantations, live, dead and resorbed fetuses, preimplantation loss rate, and postimplantation loss rate were observed in this study.

**Results:**

Obvious toxicity of male reproductive system was found at the dose of 15 mg/kg including decreases in testicular and epididymal weight, also in sperm motility rate. Whereas the increase in sperm abnormality rate was observed. However, no significant effects of FLC were found on lutea count, implantations count, fetuses count and weight, live fetuses count (rate), dead fetuses count (rate), resorbed fetuses count (rate), placentas weight, fetuses gender, preimplantation loss (rate) and postimplantation loss (rate). Furthermore, FLC had no adverse effects on fertility and early embryonic development in rats.

**Conclusion:**

The no-observable-adverse-effect level (NOAEL) of FLC on fertility and early embryonic development in rats was considered to be 5 mg/kg/day.

## Introduction

More and more studies have shown that environmental pollutants have adverse effects on fertility and embryonic development in recent years. For example, long-term exposure to organophosphorus pesticides could reduce the quality and number of sperm [1, 2], and the occupational exposure to organophosphorus pesticides could interfere with male reproductive parameters, including reduction in semen quality and changes in reproductive hormone [3]. It is generally known that pregnancy altered women’s metabolism and made them more susceptible to environmental pollutants [4], which could ultimately cause damage in female fertility [5, 6]. Thus, it is important to assess the health risks of pesticides to fertility and early embryonic development in population.

Flurochloridone (FLC) is a pyrrolidone herbicide developed by Stauffer Chemical CO. in 1970’s and put into production in 1980’s. It could lead to the accumulation of phytoene and then a decrease in the content of chlorophyll and β-carotene, through inhibition of the Phytoene desaturase (PDS), which is the key rate-limiting enzyme in carotenoid biosynthetic pathway. Sensitive plants absorbed FLC through roots, stems and coleoptiles and gradually undergonebleaching, yellowing and eventually leading to tissue necrosis [7]. FLC was included in Annex I to Directive 91/414/EEC on 1 June 2011 by European Union (EU) [8],.Due to its good effect on broad-leaved weeds, FLC has been widely used around the world [9–11].

At present, the toxicity studies of FLC mainly focus on two aspects: (1) Phytotoxicity. Considering the effect of FLC on peas [12] and sunflowers [10], it was easy to find that FLC changed the antioxidant system and the level of reactive oxygen species (ROS) in plants. This was also related to the mechanism of FLC, because it inhibited the production of carotenoids which played an important role in photoprotection by scavenging ROS in plants [13]. (2) Toxicity in rats. FLC toxicity studies indicated that the target organs were testes and epididymides of male animals, while FLC was almost non-toxic to female animals at the same dose [14, 15]. FLC could induce primary cultured sertoli cell apoptosis [16] and perturb blood-testis barrier/sertoli cell barrier function [17]. We found that the oral no-observable-adverse-effect level (NOAEL) in the 90-day subchronic toxicity test was 3 mg/kg/day in rats in our previous study [14, 18], which was an order of magnitude lower than reported by European Food Safety Authority (EFSA). This result was also consistent with our results of RNA-seq analysis in FLC-treated rat testes, which indicated that the expression of testicular RNA in the 3 mg/kg/day group was consistent with the vehicle control group.

When poisons are toxic to the reproductive system, it may be also toxic to fertility and fetal development. It was still unknown that whether the damage caused by FLC to male reproductive system could affect fertility and early embryonic development or not. Regrettably, EFSA did not specify FLC’s reproductive toxicity in the re-evaluation report adopted in 2017 [19]. Therefore, the present study of potential reproductive toxicity for FLC is very necessary. According to the results of our previous toxicity tests [18], FLC at doses of 2 mg/kg,5 mg/kg and 15 mg/kg was set to investigate the reproductive toxicity on fertility and early embryonic development in rats.

## Methods

### Experimental animals

The specific-pathogen-free (SPF) Sprague-Dawley rats, supplied by Beijing Vital River Laboratory Animal Technology Co., Ltd. (Beijing, China), were kept in animal room maintained at 23 ± 2 °C, relative humidity of 40%~ 70%, under a 12 h light/dark cycle. All rats were maintained with free access to sterile water and feed. Animal dosing and toxicology analyses were performed at the Shanghai Institute for Food and Drug Control (SIFDC, Shanghai, China). All protocols were approved by the Institutional Animal Care and Use Committee of SIFDC (IACUC-SIFDC16106).

### Materials

Flurochloridone (purity> 96.8%) was purchased from Jiangxi Anlida Chemical Co., Ltd. (Jiangxi, China). All other chemicals used were of the highest commercial grade available.

### Sample preparation procedures

FLC was suspended in 0.5% (w/v) sodium carboxymethyl cellulose (CMC-Na), a vehicle, at concentrations of 0.1 mg/mL, 0.25 mg/mL and 0.75 mg/mL and fresh samples were prepared once a week. The suspension was stirring during oral administration at room temperature.

### Experimental design

One hundred male and 100 female rats were used in this study. Males (approximately 6~8 weeks of age, weighting 187.9 g~ 230.6 g) were obtained 7 weeks earlier than females (approximately 8~9 weeks of age, weighting 168.5 g~ 202.7 g). After 7 days to adapt to the environment, rats were divided into four groups at random by body weight, namely, vehicle control group (0 mg/kg, *n* = 25), and 2 mg/kg (*n* = 25), 5 mg/kg (*n* = 25) and 15 mg/kg (*n* = 25) FLC-treated groups.

The day of first administration was defined as Administration Day 1 (AD1) and the dosage was adjusted according to body weight twice a week after each weighing. Prior to cohabitation, male and female rats in vehicle control and FLC-treated groups were intragastrically dosed for 9 and 2 consecutive weeks at a dose volume of 20 mL/kg, respectively. Then male and female rats of the same group of dosage were cohabitated in a 1:1 ratio for a period of 2 weeks in the home cage of the sexually mature breeder males.

Vaginal smear examination was conducted for female rats in the morning during the period of mating until sperm or vaginal plug was detected or till 2 weeks. This day was defined as Gestation Day 0 (GD0). Treatment lasted throughout the mating period for males and continued until GD7 for females. Pregnant rats were subjected to a caesarean section on GD13, and males were sacrificed on GD0.

### Outcomemeasures

Physical signs, behavior, and survival of rats were closely observed and recorded every day for all animals during the study period. Regularity and length of estrous cycle were examined in females.

Food consumption data were collected once a week during premating period for males and females, and also collected during gestation periods for females.

Body weight data were collected twice a week during premating period. Female rats were also weighed on GD0, GD4, GD7, GD11 and GD13.

A vaginal smear was taken daily for 2 weeks after beginning treatment to examine the regularity and length of estrous cycle in females. Estrus cycles were divided into four stages, such as proestrus, estrus, metestrus, and diestrus. One estrus cycle was defined as the period between the initiation of first estrus and before the initiation of next estrus or the initiation of first diestrus and before the initiation of next diestrus.

Terminal inspections were taken for male and female rats respectively after they were sacrificed by exsanguination while under deep anesthesia with ethylurethanm. Testes and epididymides were separated, weighed and then testes and the right epididymis were preserved in modified Davison’s fixative. Testes and epididymides were then put into 10% neutral buffered formalin 24 h after being preserved in modified Davison’s fixative, then embedded in paraffin, sectioned, stained with hematoxylin and eosin (H&E), and examined by histopathological analysis. The left epididymis was weighted, homogenized and placed in RPMI1640 containing 5% BSA (bovine serum albumin) to evaluate motility, count, and morphology of sperm. Sperm was incubated for approximately 2 to 5 min in 1% BSA- RPMI1640 culture media. Samples were placed on glass slides and evaluated for motility and total sperm counts. For motility evaluation, at least 200 static sperm were recorded. Sperm samples from each group of animals were also smeared on glass slides, and examined for malformations using a light microscopeat 200 sperm/smear.

For each female, corpora lutea count, implantation count, and numbers of fetuses/litter, live fetuses/litter, dead fetuses/litter and resorbed fetuses/litter were recorded. Number of preimplantation loss and postimplantation loss were also calculated and recorded.

### Statistical analysis

All measurements were expressed as the means±standard deviation (SD). Statistical analysis was conducted by using IBM SPSS Statistics Version20. The significant probability values are represented as asterisks, *p* < 0.05 (^*^) or *p* < 0.01 (^**^).

The variance of quantitative data was checked using Levene’s test. If *p* > 0.05, Data was subjected to one-way analysis of variance (ANOVA).On the other side, if *p* < 0.05, data was analyzed by Dunnett T3 test. If ANOVA tests showed a significant difference (*p* < 0.5), the data was analyzed by multiple comparison tests (0.05 and 0.01 levels) using Dunnett’s test. Qualitative data was checked using Kruskal-Wallis test. If *p* < 0.05, Dunnett’s test (0.05 and 0.01 levels) after rank-to-turn conversion was further used. Mating indices and pregnancy rates were subjected to the Fisher’s exactprobability test.

## Results

### Clinical observation

No animals died before being sacrificed. Physical observations indicated no obvious toxicity signs, including no changes in skin, fur, eyes mucous membrane, behavior patterns, tremors, salivation, and diarrhea of the rats. No statistically significant differences were observed in male and female food consumption among three doses of FLC-treated group and vehicle control group (*p* > 0.5).

### Body weight of male and female rats

**Fig. 1:**
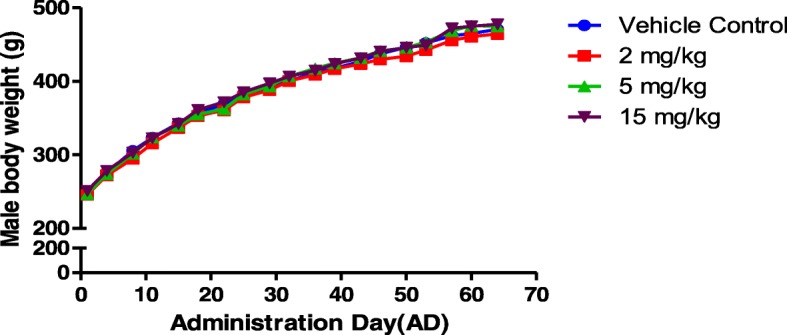
Male body weight changes during Administration Day (AD), *n* = 25 in each group

Body weight of male rats were determined during premating period. There were no statistically significant differences in male body weight between three doses of FLC-treated groups and vehicle control group (Fig. 1).

**Fig. 2:**
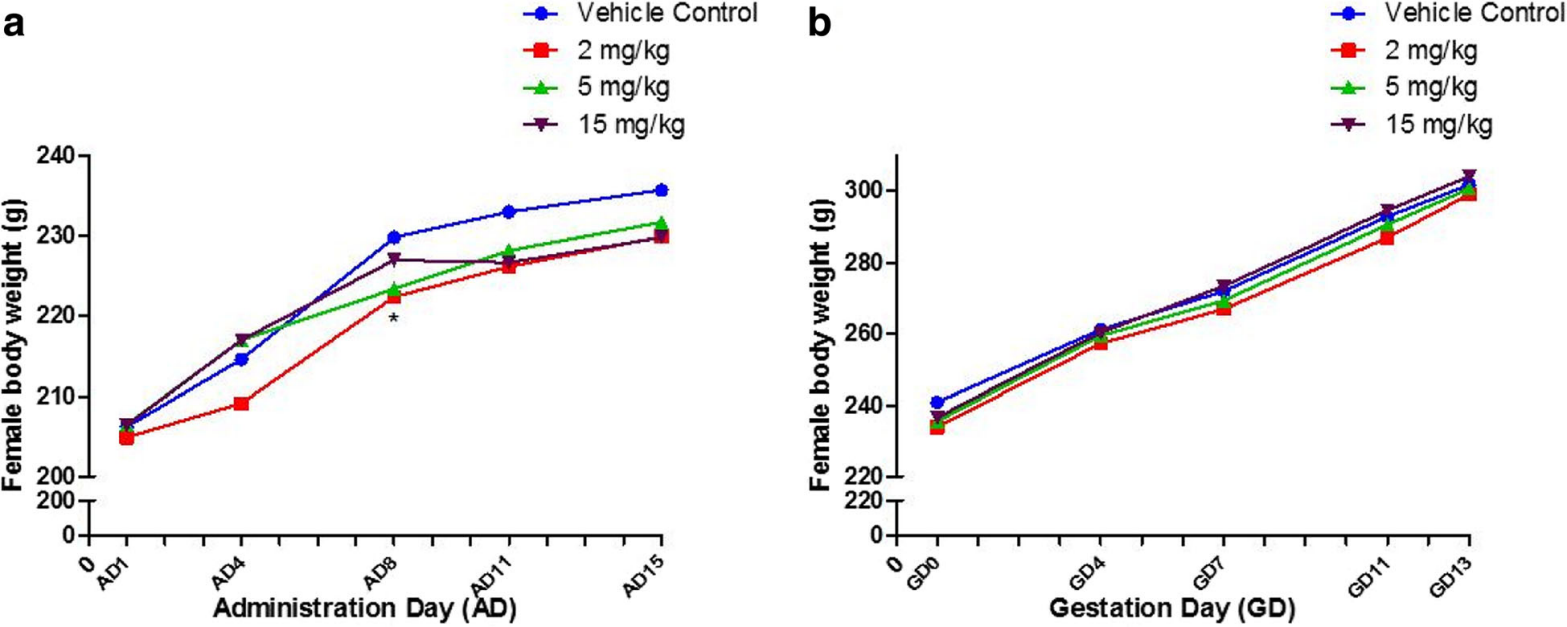
Female body weight changes. **a** Administration Day (AD), *n* = 25 in each group. **b** Gestation Day (GD), vehicle control group (*n* = 24), 2 mg/kg group (*n* = 25), 5 mg/kg group (*n* = 23) and 15 mg/kg group (*n* = 25). Note: ^*^ A significant difference at *p* < 0.05 level compared with the control at this time point

Weight and maternal weight of female rats in four experimental groups also increased time dependently. We noted that the weight of rats in 2 mg/kg group was significantly decreased compared with vehicle control group on AD8 (*p*<0.05) (Fig. 2), but this change was transient and not considered biologically relevant during continued observation of body weights in this group.

### Reproductive organs and sperm examination of male rats

Coefficients of testis and epididymis were significantly lower in 15 mg/kg group than in vehicle control group (*p* < 0.05 and *p* < 0.01), whereas no statistically significant differences were observed in weight of testis and epididymis. Sperm abnormality rate was significantly higher and sperm motility rate was significantly lower in 15 mg/kg group than in vehicle control group (*p* < 0.01). These two parameters all had a dose-dependent change (Table 1).

**Table 1:** Reproductive organs and sperm examination of male rats (mean ± SD)

Parameters	Vehicle control *n* = 25	2 mg/kg *n* = 25	5 mg/kg *n* = 25	15 mg/kg *n* = 25
Testicular weight (g)	3.3545 ± 0.3836	3.2989 ± 0.4934	3.5050 ± 0.2843	3.1803 ± 0.3067
Epididymal weight (g)	1.0619 ± 0.0985	1.0110 ± 0.1356	1.0777 ± 0.0707	0.9779 ± 0.1489
Testicular coefficient (%)	0.75 ± 0.13	0.72 ± 0.10	0.74 ± 0.07	0.68 ± 0.07^*^
Epididymal coefficient (%)	0.24 ± 0.04	0.22 ± 0.03	0.23 ± 0.02	0.21 ± 0.03^**^
Sperm count (× 10^8^/g)	1.55 ± 0.62	1.67 ± 0.61	1.58 ± 0.50	1.18 ± 0.38
Sperm abnormality rate (%)	0.37 ± 0.43	0.65 ± 0.70	0.95 ± 0.99	1.86 ± 1.65^**^
Sperm motility rate (%)	65.64 ± 9.54	66.81 ± 12.91	56.57 ± 15.02	55.13 ± 9.31^**^

^*^p < 0.05 compared with the vehicle control group

^**^p < 0.01 compared with the vehicle control group. Coefficients of organs: (organ weight/body weight) × 100

**Fig. 3:**
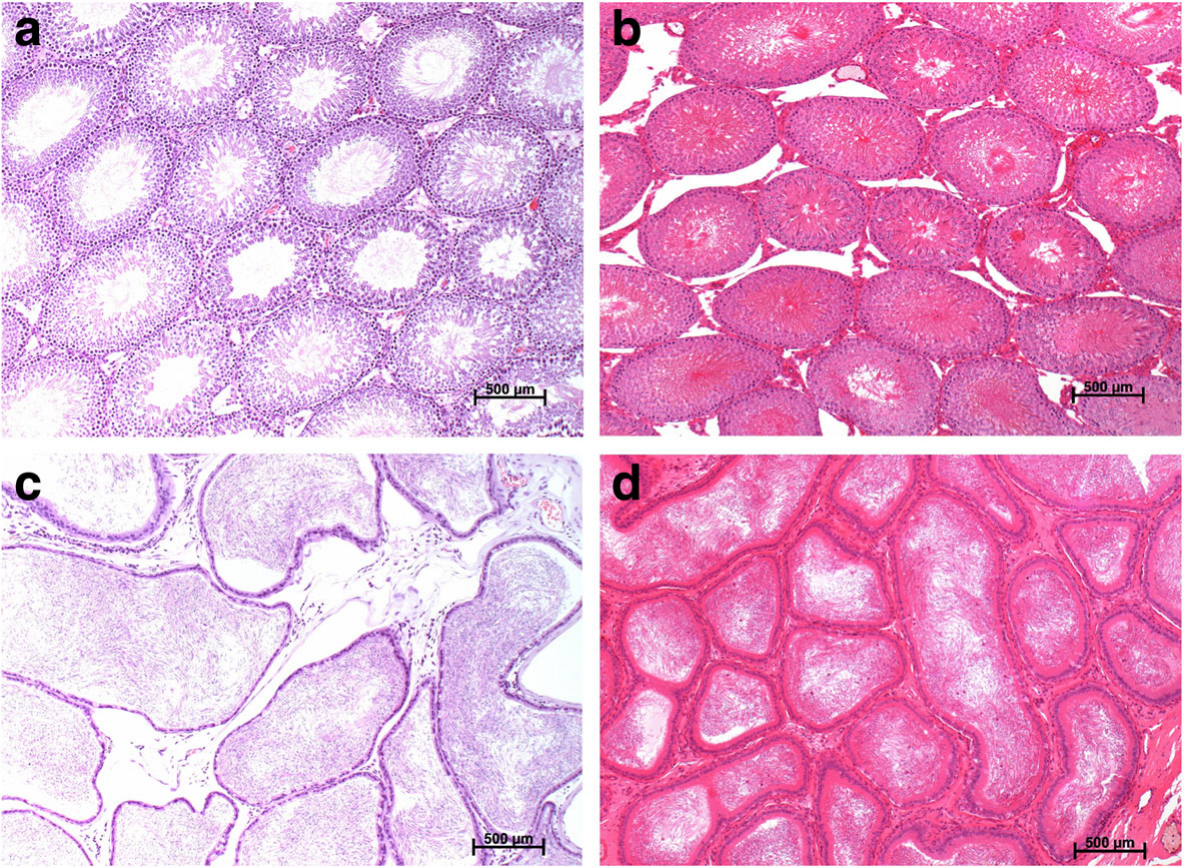
Histopathology of reproduction organs of rats (at 100 × magnification, H&E stain). **a** Testis, vehicle controlgroup; **b** Testis, 15 mg/kg group; **c** Epididymis, vehicle control group; **d** Epididymis, 15 mg/kg group

Only one rat in 2 mg/kg group was found testicular atrophy of one side by necropsy, while histopathology revealed that the testis was atrophic and calcified as a background lesion and was not associated with FLC. There were no histopathological abnormalities in other reproductive organs in the other male rats (Fig. 3).

### Effects of FLC on fertility

Overall, the copulation index, fecundity index and fertility index were similar among the vehicle control group and three doses of FLC-treated groups. FLC significantly shortened the mating period in 15 mg/kg group (*p* < 0.05) (Table 2).

**Table 2:** Effects of FLC on fertility

Sex	Parameters	Vehicle control	2 mg/kg	5 mg/kg	15 mg/kg
Male	Cohabited (n)	25	25	25	25
	Copulated (n)	24	25	23	25
	Females pregnant (n)	24	25	23	25
	Copulation index (%)	96	100	92	100
	Fecundity index (%)	100	100	100	100
	Fertility index(%)	96	100	92	100
Female	Cohabited (n)	25	25	25	25
	Copulated (n)	24	25	23	25
	Pregnant (n)	24	25	23	25
	Copulation index (%)	96	100	92	100
	Fecundity index (%)	100	100	100	100
	Fertility index (%)	96	100	92	100
	Days of mating period (d)	3.4 ± 1.4	3.3 ± 2.7	3.1 ± 1.7	2.6 ± 1.2^*^
	Days of estrous cycle (d)	4.1 ± 2.7	3.3 ± 3.3	4.3 ± 2.4	4.1 ± 1.9
	Numbers of estrous cycle (d)	1.5 ± 0.9	1.1 ± 1.1	1.6 ± 1.0	1.8 ± 1.0

Copulation index (%): (number of copulated/number of cohabited) × 100

Fecundity index (%): (number of females pregnant/number of males copulated) × 100

Fertility index (%): (number of pregnant/number of cohabited) × 100

Note: ^*^p < 0.05 compared with the vehicle control group

### Pregnancy outcome and early embryonic development in female rats

**Fig. 4:**
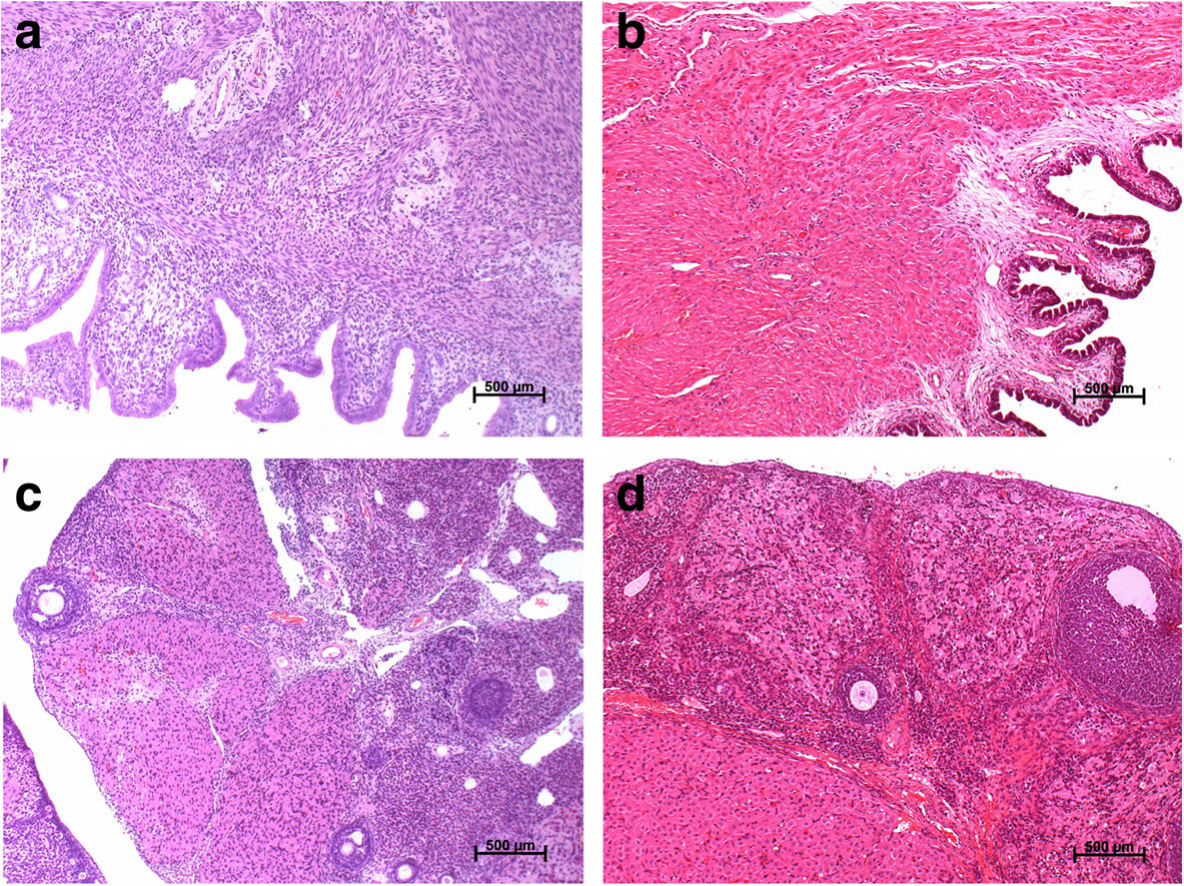
Histopathology of reproduction organs of rats (at 100 × magnification, H&E stain). **a** Uterus, vehicle control group; **b** Uterus, 15 mg/kg group; **c** Ovary, vehicle control group; **d** Ovary,15 mg/kg group

In this study, FLC had no significant adverse effects on female rats and early embryonic development. No histopathological abnormalities were observed in reproductive organs in female rats (Fig. 4).

The number of corpra lutea, implantations, fetuses, live fetuses, dead fetuses, resorbed fetuses, preimplantation loss and postimplantation loss were also not significantly influenced by FLC administration (Table 3, *p* > 0.05).

**Table 3:** Fertility and pregnancy outcome in female rats (mean ± SD)

Parameters	Vehicle control *n* = 24	2 mg/kg *n* = 25	5 mg/kg *n* = 23	15 mg/kg *n* = 25
Lutea (n)	17.5 ± 3.1	17.7 ± 2.8	18.1 ± 4.4	16.7 ± 3.4
Implantations (n)	15.4 ± 2.4	15.5 ± 1.9	14.6 ± 2.2	14.7 ± 1.4
Fetuses (n)	15.4 ± 2.4	15.5 ± 1.9	14.6 ± 2.2	14.7 ± 1.4
Live fetuses (n)	14.7 ± 2.8	14.6 ± 2.0	13.8 ± 2.6	14.1 ± 1.5
Dead fetuses (n)	0.0 ± 0.0	0.0 ± 0.0	0.0 ± 0.0	0.0 ± 0.0
Resorbed fetuses (n)	0.7 ± 1.2	0.8 ± 1.0	0.8 ± 1.0	0.6 ± 0.7
Live fetuses rate (%)	94.94 ± 10.34	94.64 ± 6.81	93.59 ± 10.48	96.02 ± 4.51
Dead fetuses rate (%)	0.00 ± 0.00	0.00 ± 0.00	0.00 ± 0.00	0.00 ± 0.00
Absorbed fetuses rate (%)	5.06 ± 10.34	5.36 ± 6.81	6.41 ± 10.48	3.98 ± 4.51
Preimplantation loss (n)	2.1 ± 3.6	2.2 ± 2.5	3.5 ± 5.4	2.0 ± 3.0
Preimplantation loss rate (%)	17.04 ± 35.94	15.04 ± 17.41	29.65 ± 60.55	13.58 ± 19.06
Postimplantation loss (n)	0.7 ± 1.2	0.8 ± 1.0	0.8 ± 1.0	0.6 ± 0.7
Postimplantation loss rate (%)	5.06 ± 10.34	5.36 ± 6.81	6.41 ± 10.48	3.98 ± 4.51

## Discussion

The concern that exposure to pesticides affects reproductive system and leads to infertility has been a focus in recent decades [20]. Populations in developing and under-developed countries are reportedly more vulnerable to these pesticides [21]. The globally harmonized system of classification and labeling of chemicals (GHS) showed the reproductive toxicity of FLC was classified as suspected of damaging fertility or the unborn child (level 2, no supporting data available) [15]. Moreover, our previous subchronic toxicity test noted that FLC caused decreases in testicular weight and serum testosterone. Sperm abnormalities in epididymis were also found increased at 10 mg/kg/day [18]. Thus, the toxic effect of FLC on fertility and early embryonic development was investigated in this study.

In the present study, FLC had no obvious effects on the physical signs, animal behaviors, survival rate, food consumption and body weight. The data obtained about the effects of FLC on fertility and early embryonic development was considered to be scientific at these doses.

In rodent-based toxicology studies involving gonadal effects testicular weight is a required measure, complementing histopathologic findings. The entire stage of spermatogenesis is sensitive to foreign poisons. Sperm count, sperm motility rate and sperm abnormality rate are important indicators for evaluating the toxicity of poisons on male reproductive system. In our study, the coefficients of testis and epididymis was decreased with FLC in 15 mg/kg group. As no histopathological abnormalities were found in testes and epididymides, the decreased weight of testes and epididymides may be also associated with the decreased sperm [22]. In consistent with Katoh et al.’s study, our result showed that FLC also decreased sperm motility and increased sperm abnormality rate, while these two parameters all had dose-dependent changes. In the present study, male rats were dosed FLC for 63 days, a complete cycle of sperm development, which could fully demonstrate the toxicity of FLC on sperm. These data suggested that FLC can be toxic to sperm before it caused histopathological abnormalities to testes and epididymides. Sperm was sensitive to FLC.

There were several explanations about the mechanisms of FLC effects upon the decrease in sperm quality. First, FLC could adversely affect sperm quality through spermatogenic cells. Our previous studies found that FLC could cause apoptosis in co-cultured sertoli-germ cells by intrinsic apoptotic signaling pathway with the assistant of NFκB and p38MAPK pathways [23]. In the 90-day toxicity study, the number of spermatogenic cells in the seminiferous tubules was decreased, and in the high dose group, sperm cells disappeared [14].

Second, the decrease in sperm quality may be related to the effects of FLC on sertoli cells. Liu et al. [16] found that FLC could lead to apoptosis and induce the elevation of ROS levels, intracellular calcium levels, and ERK1/2 activation in sertoli cells. FLC could also perturb blood-testis-barrier (BTB)/sertoli cell barrier function through Arp3-mediated F-actin disorganization [17]. Xu et al. [23] reported that FLC disrupted the structural integrity of BTB, impaired the paracrine function of sertoli cells, resulting in the insufficient secretion of testosterone and iron required for the differentiation and growth of sperm in the seminiferous tubules. In the present study, although no changes in testicular pathology were observed, coefficient of testis was decreased in 15 mg/kg group, which indicated that FLC may had slightly adverse effect on testes at this dose.

Third, the decrease in sperm quality may be related to the effects of FLC on epididymides. Perfluorooctanoic acid (PFOA) could reduce epididymal weight and then lead to sperm injury [24]. Sperm gains athletic ability and maturity in epididymides. In Zhang et al.’s study, rat epididymides was severely atrophied, and no mature sperm was found, indicating that FLC may damage epididymides and disturbed sperm maturation [14]. In consistent with this study, our result showed that coefficient of epididymis was significantly decreased, while sperm abnormality rate was significantly increased and sperm motility rate was decreased.

Last but not the least, the decrease in sperm quality in FLC treated rats may be related to oxidative stress. In vivo, FLC can be conjugated with glutathione (GSH) which is an antioxidant that prevents damage to important cellular components caused by ROS. GSH had been shown in rat sperm mitochondria to play a significant role in peroxyl scavenging mechanism and in maintaining sperm motility [25]. Abundant conjugation with FLC may lead to GSH depletion and correspondingly result in increased oxidative stress [26] which may lead to an imbalance between ROS generation and antioxidant scavenging activities [27]. Furthermore, high levels of ROS caused by GSH depletion were potentially toxic to sperm quality and function [28], it damaged cell membranes and inhibited sperm motility and likely caused sperm DNA fragmentation [29].The above results indicated that FLC effected testes and epididymides, which in turn disturbed the main stages of sperm formation, and then decreased the sperm quality.

Although FLC caused damage to the sperm, there was no change in mating rate and pregnancy rate in each dose group in our study, probably because the number of sperm produced by rodents far exceeded the minimum requirements for fertility. It was found that Sperm quality played an important role in determining human fertility [30, 31]. Decreased sperm motility and morphology were significant predictive factors for high sperm DNA damage [29], as in vivo fecundity decreased progressively when > 30% of the sperm were identified as having DNA damage [28]. Therefore, it is hard to say FLC have no adverse effects on human fertility, and population data is needed for proof.

For female rats, FLC had no significant effects on lutea count, implantations count, fetuses count and weight, live fetuses count (rate), dead fetuses count (rate), resorbed fetuses count (rate), placentas weight, fetuses gender, preimplantation loss (rate) and postimplantation loss (rate). FLC probably had no effects on fertility and early embryo in female rats. However, this conclusion was only suitable for negative results when rodents were used in fertility research. If there was a positive result, other studies were needed to be added. As FLC had adverse effects on reproductive system of male rats, the effects of FLC on human fertility and pregnancy outcomes need further research.

## Conclusion

FLC had obvious toxicity to male reproductive system at the dose of 15 mg/kg with decreases of testicular weight, epididymal weight and sperm motility rate, also an increase in sperm abnormality rate. FLC had no adverse effect on fertility and early embryonic development in rats. The NOAEL of FLC on fertility and early embryonic development in rats was considered to be 5 mg/kg/day.

## Data Availability

The dataset supporting the conclusions of this article is included within the article.

## Abbreviations

AD: Administration Day
ANOVA: One-way analysis of variance
BSA: Bovine serum albumin
EFSA: European Food Safety Authority
EU: European Union
FLC: Fluorochloridone
GD: Gestation Day
GHS: Classification and labeling of chemicals
H&E: Hematoxylin and eosin
NOAEL: No-observable-adverse-effect level
PDS: Phytoene desaturase
PFOA: Perfluorooctanoic acid
ROS: Reactive oxygen species
SIFDC: Shanghai Institute for Food and Drug Control
SPF: Specific-pathogen-free
